# Methylome of human skeletal muscle after acute & chronic resistance exercise training, detraining & retraining

**DOI:** 10.1038/sdata.2018.213

**Published:** 2018-10-30

**Authors:** R. A. Seaborne, J. Strauss, M. Cocks, S. Shepherd, T. D. O’Brien, K. A. van Someren, P. G. Bell, C. Murgatroyd, J. P. Morton, C. E. Stewart, C. A. Mein, A. P. Sharples

**Affiliations:** 1 Institute for Science and Technology in Medicine (ISTM), School of Medicine, Keele University, Staffordshire, United Kingdom; 2 Research Institute for Sport and Exercise Sciences, Liverpool John Moores University, Liverpool, United Kingdom; 3 Centre for Genomics and Child Health, Blizard Institute, Barts and the London School of Medicine and Dentistry, Queen Mary University of London, London, UK; 4 Department of Sport, Exercise and Rehabilitation, Northumbria University, Newcastle upon Tyne, United Kingdom; 5 School of Healthcare Science, Manchester Metropolitan University, Manchester, United Kingdom; 6 The Genome Centre, Blizard Institute, Barts and the London School of Medicine and Dentistry, Queen Mary University of London, London, UK

**Keywords:** Physiology, DNA methylation

## Abstract

DNA methylation is an important epigenetic modification that can regulate gene expression following environmental encounters without changes to the genetic code. Using Infinium MethylationEPIC BeadChip Arrays (850,000 CpG sites) we analysed for the first time, DNA isolated from untrained human skeletal muscle biopsies (vastus lateralis) at baseline (rest) and immediately following an acute (single) bout of resistance exercise. In the same participants, we also analysed the methylome following a period of muscle growth (hypertrophy) evoked via chronic (repeated bouts-3 sessions/wk) resistance exercise (RE) (training) over 7-weeks, followed by complete exercise cessation for 7-weeks returning muscle back to baseline levels (detraining), and finally followed by a subsequent 7-week period of RE-induced hypertrophy (retraining). These valuable methylome data sets described in the present manuscript and deposited in an open-access repository can now be shared and re-used to enable the identification of epigenetically regulated genes/networks that are modified after acute anabolic stimuli and hypertrophy, and further investigate the phenomenon of epigenetic memory in skeletal muscle.

## Background Summary

A hallmark of eukaryotic cells is their ability to alter the expression of their inherited genetic code, in order to adapt to external stimuli in an attempt to maintain functional homeostasis. In this regard, epigenetics, referring to heritable changes in gene function that are not due to changes in the DNA sequence itself^1^, is a mechanism by which modifications at specific genomic sites (predominantly chromatin and DNA) have the ability to alter fundamental DNA-based processes including transcription, translation and DNA repair. DNA methylation is an epigenetic mark that, via specific enzymes, incorporates a methyl group to the fifth position of the pyrimidine ring of a cytosine (5mC) in a cytosine-guanine base pairing (CpG), attenuating the performance of transcriptional apparatus and producing a reduction in the expression of specific gene transcripts^2^.

Seminal *in-vivo* work in human skeletal muscle showed that acute aerobic exercise (80% VO_2_ max until 1674 kj/400 kcal was expended) rapidly alters promotor DNA methylation in important mitochondrial related transcripts (PGC1*α*, PDK4, TFAM and PPAR*δ*), and was associated with a time-dependent increase in their transcript expression, work that added to previous insights into the role of DNA methylation during aerobic based interventions^3,4^. The same authors further confirmed these findings, where electrically stimulated (a set equated to 0.3 s trains of 25 Hz, 0.1 ms impulses, repeated every second for 5-mins, with sets repeated every 10-mins for 1 h) mouse soleus muscle produced significant reductions in promotor methylation (hypomethylation) 45-min post stimulation, that was met with significant increases in gene expression of PGC1*α*, PDK4 and PPAR*δ* 180-min later^5^. Similar findings having since been replicated in rodents, where mice subjected to an acute bout of endurance exercise (35 rpm on a 3.0 cm rotarod machine, increasing to 45 rpm over a 60-min period), displayed increased hydroxy-methylation of the PGC1*α* promotor that correlated to increased basal mRNA expression^6^. Furthermore, moderate intensity exercise has also been shown to produce alterations in promotor methylation, where 120-mins of cycling (60% VO_2_ max) resulted in hypermethylation of *FABP3 and COX4L1* creating a corresponding reduction in gene expression in healthy adult human subjects^7^. Collectively, these data suggest that changes in promotor methylation are associated with alterations in gene transcript expression following aerobic exercise. However, at present there are a limited number of studies that investigate the effects of resistance exercise on DNA methylation in skeletal muscle tissue. Currently, there have been studies investigating changes in methylation of leukocyte DNA following 8-weeks of resistance exercise in healthy male subjects^8^. Following confirmation that resistance exercise induced large scale methylome and transcriptome alterations in the leukocytes, the authors identified two key anabolic transcripts, growth hormone-releasing hormone (GHRH) and fibroblast growth hormone 1 (FHG1), as displaying an inverse relationship, where reductions in methylation were associated with enhanced gene expression^8^. In skeletal muscle, the epigenome was examined following a short-term period (9 days) of either high-fat feeding or high-fat feeding plus resistance exercise, displaying a preferential hypermethylated profile following high-fat feeding and a hypomethylated signature following high-fat feeding plus resistance exercise^9^. Closer analysis identified two transcripts that showed a distinct inverse relationship between gene expression and promotor DNA methylation following high-fat feeding plus resistance exercise^9^. Despite these findings, and further reports identifying the importance of promotor DNA methylation in muscle mass regulation following muscle atrophy^10^, and studies suggesting muscle cells retain DNA methylation over several daughter populations when encountering early proliferative-life inflammation (TNF-alpha)^11,12^, there is distinct lack of current research elucidating the DNA methylation changes in skeletal muscle tissue using the latest genome-wide array techniques following acute resistance exercise alone or after chronic resistance exercise. There are also currently no studies investigating the effect of detraining or later retraining on the skeletal muscle methylome in humans.

However, in a recent multi-stage, *in-vivo* within-subjects experiment, eight healthy previously un-trained male subjects performed an acute bout of resistance exercise (acute RE), followed by a progressive 7-week resistance training programme (training) to evoke muscle hypertrophy. Subjects then completely ceased exercise for a period of 7-weeks to return muscle size back towards pre-exercise levels (detraining), followed by a further and final period of 7-weeks resistance exercise to evoke hypertrophy (retraining). Fasted biopsies were obtained at baseline and post-acute RE, training, detraining and retraining. DNA methylation was detected via the use of the Infinium^®^ MethylationEPIC BeadChip Array (Illumina, Inc., California, United States) that analyses over 850,000 CpG sites of the human epigenome. The EPIC BeadChip provides dense coverage of gene regions including CpG islands, RefSeq genes, ENCODE transcription factor binding sites and FANTOM5 enhancers. Furthermore, the relatively new array technology covers over 90% of the previous array model (HumanMethylation 450 K BeadChip, Illumina, United States), with an added 350,000 CpG sites of regions identified as potential enhancers by FANTOM5^13^ and ENCODE^14^. Collectively, the array provides one of the most comprehensive but discernible explorations of known CpG sites in the human genome^15^. Thus, utilising this array in our experimental model, we provide for the first time, a comprehensive and extensive data descriptor of the human methylome after both acute and chronic resistance exercise training, detraining and retraining.

In the primary research paper^16^, in which the present manuscript serves as a data descriptor, we identified that 30 min following a single/acute bout of resistance exercise (acute RE), resulted in extensive modifications to the human methylome, where 17,884 CpG sites were significantly modified (P ≤ 0.05), preferentially favouring reduced DNA methylation (hypomethylation), where 10,284 CpG sites were hypomethylated versus 7,600 CpG sites that were hypermethylated. Furthermore, we reported large alterations in DNA methylation patterns following chronic resistance exercise induced hypertrophy (6.5 ± 1% increase in lower limb lean mass vs. baseline) with 17,365 significantly differentially modified CpG sites (9,153 hypomethylated and 8,212 hypermethylated). Followed by detraining where muscle returned back to baseline levels after complete cessation of resistance exercise with 17,529 significantly modified CpG sites (8,891 hypomethylated and 8,636 hypermethylated) and finally a subsequent period of retraining induced hypertrophy (12.4 ± 1.3% increase in lower limb mass vs. baseline and +5.9% ± 1% vs. the earlier period of training induced hypertrophy) where 27,155 CpG sites were significantly modified (18,816 hypomethylated and 8,339 hypermethylated). Demonstrating the largest increase in lean mass of the lower limbs during retaining was associated with the largest increase in hypomethylation of the genome (18,816 CpG sites significantly modified after later retraining versus 9,153 CpG sites after earlier training). Resulting in 69% of the differentially modified CpG sites during retaining favouring hypomethylation versus hypermethylation. We identified a number of genes (AXIN1, GRIK2, CAMK4, TRAF1) as hypomethylated at the DNA level that also demonstrated enhanced gene expression after training. These genes also maintained their hypomethylated status even during detraining where muscle mass returned to control levels, indicating a memory of these genes methylation signatures following earlier training induced hypertrophy. Further, we identified several genes (UBR5, RPL35a, HEG1, PLA2G16, SETD3) that displayed hypomethylation and enhanced gene expression following earlier training induced hypertrophy, positively correlating with lean mass increases, with some of these genes demonstrating the largest increases in hypomethylation, gene expression and muscle mass after later retaining induced hypertrophy, indicating an epigenetic memory in these genes. Finally, there were also a group of epigenetically ‘sensitive’ genes (GRIK2, TRAF1, BICC1, STAG1) following acute resistance exercise demonstrating hypomethylation after a single bout of exercise that was maintained 22 weeks later with the largest increase in gene expression and muscle mass after retraining. Overall, we identified an important epigenetic role for a number of largely unstudied genes in muscle hypertrophy/memory. In the present manuscript we provide a detailed description of the methodological approach used to analyse this genome wide DNA methylation data set in doing so, we hope to encourage the wider data sharing and reuse use of this valuable data set for future comparative/bioinformatic analysis investigating the epigenetic mechanisms of skeletal muscle adaptation and memory.

## Methods

### Human Participants and Ethical Approval

Ethical approval for the study was granted by the NHS West Midlands Black Country, UK, Research Ethics Committee (NREC, UK approval no. 16/WM/0103). Eight healthy males gave written, consent to participate in the study, following successful completion of a readiness to exercise questionnaire and a pre-biopsy screening as approved by a physician. One participant withdrew from the study at experimental week 17 of 21, for reasons unrelated to this investigation. However, consent allowed samples to be analysed prior to withdrawal, therefore for this participant all conditions were included except the final retraining condition.

### Experimental Design

**Figure 1:**
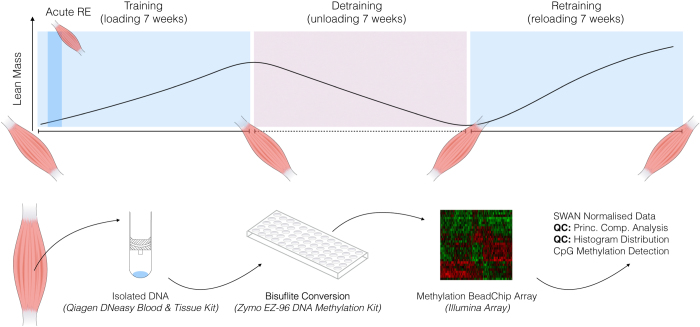
Schematic representation of human resistance exercise study and methylome-wide bioinformatics pipeline. Muscle biopsies were obtained in previously untrained male participants following 7-weeks of chronic resistance training (loading), 7-weeks exercise cessation/detraining (unloading) and a later 7-week period of chronic retraining (reloading). A biopsy was also obtained following the first acute (single) bout of resistance training (acute RE). The schematic shows the work-flow utilised to generate genome-wide DNA methylation profiles in human participants, the methodological approach and the assays utilised. Bioinformatical analysis was performed in Partek Genomic Suite (Partek Genomic Suite, V6.6, Missouri, USA). Schematic adapted from our primary paper^16^. Licensed under the Creative Commons Attribution 4.0 International Public License. To view a copy of this license, visit https://creativecommons.org/licenses/by/4.0/legalcode.

Using a within subject design eight previously untrained male participants (27.6 ± 2.4 yr, 82.5 ± 6.0 kg, 178.1 ± 2.8 cm, means ± SEM) completed an acute bout of resistance exercise (acute RE), followed by 7 weeks (3d/week) of resistance exercise (training), 7 weeks of exercise cessation (detraining) and a further period of 7 weeks (3d/week) resistance exercise (retraining). Graphical representation of experimental design is provided in Fig. 1. Whole-body fan beam dual-energy x-ray absorptiometry (DEXA), strength of the quadriceps via dynamometry and muscle biopsies from the vastus lateralis for RNA and DNA isolation were obtained at baseline, after 7 weeks training (beginning of week 8), 7 weeks detraining (end of week 14) and 7 weeks retraining (beginning of week 22), as described in our original paper^16^. A muscle biopsy was also obtained 30 min after acute RE prior to 7 weeks resistance exercise/training. Genome-wide analysis of DNA methylation was performed via Illumina EPIC array (> 850,000 CpG sites- detailed below) for participants across all conditions (*n* = 8 baseline, acute, training, detraining, *n* = 7 retraining).

### Acute resistance exercise, training, detraining and retraining

Descriptive details of exercise and biopsy procedures are taken from our primary paper^16^. Briefly, untrained male subjects initially performed an exercise familiarization week, in which participants performed all exercises with no/low load to become familiar with the exercise movements (detailed below). In the final session of the familiarization week, the load that participants could perform 4 sets of 8-10 repetitions for each exercise was assessed. Due to participants being un-customized to resistance exercise, assessment was made on competence of lifting technique, range of exercise motion and verbal feedback. Subsequently, starting load was set for each participant on an individualised basis. Three to four days later, participants then undertook a single bout of lower limb resistance exercise (acute RE) followed by biopsies 30-mins post exercise. Following this single bout of acute RE they then began a chronic resistance exercise program (training), completing 60-min training sessions (Monday-Wednesday-Friday), for 7 weeks, with 2 sessions/week focusing on lower limb muscle groups (Monday and Friday) and the third session focusing on upper body muscle groups (Wednesday). Lower limb exercises included; behind head barbell squat, leg press, leg extension, leg curl, Nordic curls, weighted lunges and calf raises. Upper limb exercises included, flat barbell bench press, machine shoulder press, latissimus dorsi pull down, bent over dumbbell row and triceps cable extension. To ensure progression in participants with no previous experience of resistance exercise, a progressive volume model was adopted^17^ in which investigators regularly assessed competency of sets, reps and load of all exercises. Briefly, exercises were performed for 4 sets of 10 reps in each set, ~90-120 s in between sets and ~3 mins between exercises. When participants could perform 3 sets of 10 repetitions without assistance and with the correct range of motion, load was increased by ~5-10% in the subsequent set and participants continued on this new load until further modification was required. Where subjects failed to complete 10 full repetitions (usually for their final sets), they were instructed to reduce the load in order to complete a full repetition range for that set or the subsequent set. Total weekly volume load was calculated as the sum of all exercise loads;

Totalexercisevolume(kgs)=(Exerciseload(kgs)*No.ofReps)*No.Sets

Load data following single acute RE stimulus and training, detraining and retraining are included in detail in our original paper^16^. Training and retraining programs were conducted in an identical manner, with the same exercises, program layout (same exercises on same day), sets and repetition pattern, as well as rest between sets and exercises. During the 7-week detraining phase, participants were instructed to return to habitual pre-intervention exercise levels and not to perform any resistance training. Regular verbal communication between researcher and participant ensured subjects followed these instructions. A trainer was present at all resistance exercise sessions to enable continued monitoring, provide verbal encouragement and to ensure sufficient progression. No injuries were sustained throughout the exercise intervention.

### Muscle Biopsies and Sample Preparation

At baseline, 30- mins post-acute resistance exercise (RE) and after 7 weeks training (beginning of week 8), 7 weeks detraining (end of week 14) and 7 weeks retaining (beginning of week 22) (Fig. 1), a conchotome muscle biopsy was obtained at rest from the vastus lateralis muscle of the quadriceps from each participant, avoiding areas of immediate proximity to previous incisions. Muscle biopsy procedures were performed following an overnight fast. Tissue samples were carefully dissected using a sterile scalpel on a sterile petri-dish, where, in the unlikely event of any fat, fibrous or other stroma tissue, samples were removed of these. Tissue samples were subsequently immediately snap frozen in liquid nitrogen before being stored at −80 °C for later DNA isolation.

### Isolation of DNA and Bisulfite Treatment

All samples were isolated using a commercially available DNA isolation kit (DNeasy Blood and Tissue Kit; Qiagen, United Kingdom) in accordance with manufacturers’ instructions. Samples were collected from −80 °C and immediately kept on ice before being immersed in 200 μl of DNA homogenate buffer (180 μl of ATL buffer and 20 μl of proteinase k) and homogenised at 6,000 rpm 40-secs (MagNA Lyser Instrument, Roche Life Sciences, UK). This step was repeated 3 times with 5 min on ice between homogenisations, to ensure total disruption of muscle cells, and the release of genomic DNA while avoiding its degradation. Suspension was supplemented with 200 μl of AL buffer before being incubated at 56 °C for 10-mins. Molecular grade ethanol (200 μl of >96% pure) was subsequently added to the suspension before being briefly vortexed and aliquoted into spin columns placed in 2 ml collection tubes (DNeasy Mini Spin Colum, DNeasy Kit, Qiagen). Spin columns were centrifuged at 6,000 g for 1-min, flow through discarded and 500 μl of buffer AW1 added to the spin column and centrifuged at 6,000 g for 1-min. Buffer AW2 was then added to the spin column and centrifuged at 20,000 g for 3-min. All flow through was discarded and spin columns were placed in new RNA/DNA free tubes (RNase-free Microfuge Tubes, Ambion^TM^, ThermoFisher Scientific, United States), before 50 μl of elution reagent (buffer AE) was added directly to the spin column and centrifuged at 6,000 g for 1-min. This elution step was repeated to yield a total DNA suspension of 100 μl per original sample. Eluted suspension was then analysed for quality and quantity (Supplementary Table 1) via ultra violet spectroscopy (Nanodrop 2000, ThermoFisher Scientific, United States).

For bisulfite conversion (EZ DNA Methylation Kit, Zymo Research, CA, United States), 5 μl of M-Dilution Buffer was added to 500 ng of isolated DNA, and distilled water (dH_2_0) was added where applicable, to yield a total suspension of 50 μl, before samples were incubated for 15-mins at 37 °C. In each sample 100 μl of prepared CT Conversion Reagent was added and incubated in the dark overnight (16-hrs) at 50 °C. Following this incubation, samples were stored on ice for 10-mins. Four-hundred μl of M-Binding Buffer was added to each well, before experimental samples were added and mixed. Suspension was centrifuged at 3,000 g for 5-mins, flow-through was discarded and 500 μl of M-Wash Buffer was added and centrifuged at 3,000 g for 5-mins. M-Desulphonation Buffer (200 μl) was added to the suspension and left to incubate at room temperature for 15-20-mins before being centrifuged at 3,000 g for 5-mins. Two wash buffer stages were then performed, where 500 μl of M-Wash Buffer was added to the suspension and centrifuged at 3,000 g for 5 and 10-mins, respectively. To elute the sample, the silicon plate was placed onto an Elution Plate and 30 μl of M-Elution Buffer was added directly to the matrix of each well and spun at 3,000 g for 3-mins.

#### Infinium MethylationEPIC BeadChip Array Amplification

All methylome wide experiments were performed in alignment with Illumina manufacturer instructions for the Infinium MethylationEPIC BeadChip Array. All samples were treated identically and in the same work flow to avoid confounding batch effects. Four μl of bisulfite converted DNA (BCD) was transferred from the bisulfite conversion plate in to corresponding wells of the MSA4 plate, where 20 μl of MA1 and 4 μl of 0.1 N NaOH were added before plate was vortexed (1600 rpm for 1-min), pulse centrifuged (280 g) and left to incubate at RT for 10-mins. Samples within this place then had 68 μl of RPM and 75 μl of MSM added before a further round of vortex and centrifugation was performed (identical to above). Samples were subsequently left in a 37 °C hybdrization oven overnight (20-24 hrs) to allow for amplification.

#### Fragmentation, Precipitation and Resuspension of Amplified DNA

FMS (50 μl) was added to each well of the MSA4 before being vortexed (1600 rpm for 1-min), centrifuged (280 g) and incubated (37 °C for 1-hr) to fragment DNA. An endpoint fragmentation was used to avoid over-fragmentation. Following incubation, 100 μl of PM1 and 300 μl of 2-propanol were added to each well interspersed with vortexing (1600 rpm for 1-min), incubation (37 °C for 5-mins) and centrifugation (280 g for 1-min). MSA4 plate was subsequently mixed via inversion (at least 10 times) incubated at 4 °C for 30-mins and centrifuged at 3000 g at 4 °C for 20-mins. The subsequent supernatant liquid was decanted out of wells and left at room temp for 1 h to dry the residual pellet. Following precipitation, each pellet in each well was resuspended in 46 μl of RA1 and incubated at 48 °C for 1-h. Finally, each resuspended pellet was vortexed for 1-min (at 18000 rpm) and pulse centrifuged at 280 g.

#### Hybdrization to BeadChip, Extension and Staining

Fragmented DNA residing on the MS4A plate was incubated at 95 °C for 20-mins to denature experimental samples before being left to stand at room temp for 30-mins and pulse centrifuged at 280 g. DNA was subsequently prepared for transfer and precisely loaded onto a working BeadChip. BeadChips were loaded into the Illumina Hyb Chamber and placed for over-night incubation at 48 °C for 16-hrs, before being washed (gentle agitation in 200 ml of PB1) and readied for BeadChip staining. Assembled flow-through chambers were loaded into a chamber rack where single based-extension occurred of each flow through assembly. This single base-extension was performed at 44 °C via the addition of the following reagents: 150 μl of RA1 with incubation of 30-secs (repeated 5 times), 450 μl of XC1, 450 μl of XC2, 200 μl of TEM and 450 μl of 95% formamide/1 mM EDTA (repeated). Each flow-through assembly was incubated for 5-mins, before 450 μl of XC3 was added (repeated). Staining of assemblies was performed in 5 repeated cycles of the following: addition of 250 μl of STM to each flow-through assembly (10-mins incubation), 450 μl of XC2 incubated for 1-min (repeated) and left to stand for 5-mins. To wash staining reagents, BeadChips were gently submerged and agitated initially in PB1 (310 ml per 8 BeadChips), and then in XC4 (same total amount of reagent), with a 5-min delay in between the use of both cleaning buffers. Finally, washed BeadChips were left to dry for 50-55-mins before being taken for BeadChip imagery, using the Illumina iScan® System (Illumina, United States).

**Figure 2:**
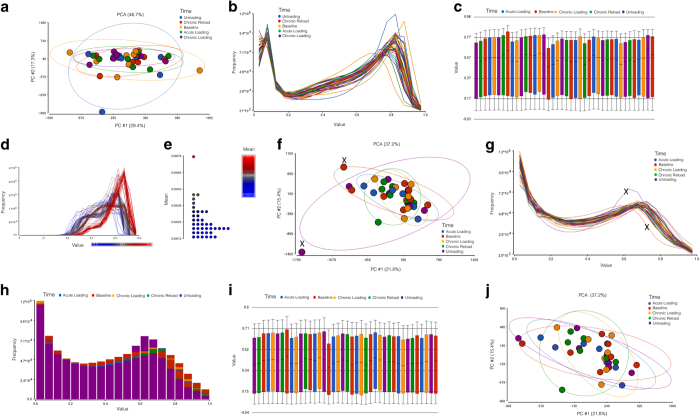
Complete quality control analysis completed on methylome-wide array. (**a**). Principal component analysis of un-normalized samples where each sample is represented by a dot. Ellipsoids have been created as 2SD’s from the centroid for each time/condition. (**b**). Frequency plot by lines, with beta values are on the horizontal axis and their frequencies on the vertical axis (un-normalised samples). (**c**). Distribution of beta values across the samples and conditions by a box-and-whiskers plot (un-normalized samples) and conditions by a box-and-whiskers plot (un-normalized samples). (**d**). Density plot of the raw probe intensities/signals after normalization demonstrating all unmethylated and methylated signals were above 11.5. (**e**). Plot of detection P values (samples normalized) with an average of 0.0000257, well below 0.01 recommended. (**f**). Principal component analysis (PCA) (samples normalized) where each sample is represented by a dot. Ellipsoids have been created as 2SD’s from the centroid for each time/condition. PCA was derived and adapted from original analysis in our primary paper^16^. Licensed under the Creative Commons Attribution 4.0 International Public License. To view a copy of this license, visit https://creativecommons.org/licenses/by/4.0/legalcode. (**g**). Frequency plot by lines, with beta values are on the horizontal axis and their frequencies on the vertical axis, representing the distribution of intensity for each probe (samples normalised) Two samples were identified as 2SD’s from the centroid (in Fig. 2A above) and as showing differential distribution patterns and were removed from further down-stream analysis (annotated via ‘X’). Histogram derived and from original analysis in our primary paper^16^. Licensed under the Creative Commons Attribution 4.0 International Public License. To view a copy of this license, visit https://creativecommons.org/licenses/by/4.0/legalcode. (**h**). Frequency plot by bars, with beta values are on the horizontal axis and their frequencies on the vertical axis (samples normalized). (**i**). Distribution of beta values across the samples and conditions by a box-and-whispers plot (samples normalized). (**j**). PCA with ellipsoids 2SD’s from the centroid for time/condition, after removal of two samples that were 2SD’s from the centroid (in Fig. 2A above) and as showing differential distribution patterns (Fig. 2B above). Baseline = biopsy at rest, Acute Loading = Acute RE, Chronic Loading = Training, unloading = Detraining, Chronic Reload = Retraining.

Following DNA methylation quantification via MethylationEPIC BeadChip array, raw IDAT files were processed on Partek Genomic Suite V.6.6 (Partek Inc. Missouri, USA). Upon import of the data, the MethylationEPIC_v-1-0_B2.bpm manifest file was used to filter out known cross-reactive probes and SNPs. Prior to normalisation we undertook principle component analysis (PCA) (Fig. 2a), density plots (Fig. 2b) and box/whisker plots (Fig. 2c) of the un-normalised samples. Following this, background normalisation was performed via the Subset-Quantile Within Array Normalisation (SWAN) method, as previously described^18^, to generate ß-values for CpG DNA methylation at >850,000 loci of the human epigenome. We also undertook density plots of the raw intensities/signals of the probes (Fig. 2d) that demonstrated all methylated and unmethylated signals were over 11.5, and the the difference between median methylation and median unmethylated signal was less than 0.5 as suggested in^19^. We also calculated the average detection p-value (0.0000257 across all samples; Fig. 2e), which was below that recommended in the Oshlack workflow, of 0.01^19^. Following normalisation, we undertook principle component analysis (PCA) (Fig. 2f), density plots by lines (Fig. 2g) and by bars (Fig. 2h) as well as box and whisker plots (Fig. 2i) of normalised samples. Outlier samples were detected using principle component analysis (PCA) and analysing the normal distribution of ß-values. Outliers were then removed if they fell outside 2 standard deviations (SDs) from the centroid (in Fig. 2f) using ellipsoids as well as showing different distribution patterns to the samples of the same condition (Fig. 2g).

## Data Records

Raw IDAT files for MethylomeEPIC BeadChip of the human epigenome were deposited in the publicly available functional genomics data repository, *Gene Expression Omnibus* (*GEO*) and have been assigned GEO accession and reference number GSE114763 (Data Citation 1).

## Technical Validation

### Validation of *In-Vivo* Changes in Muscle Morphology

As skeletal muscle samples were obtained from *in-vivo* milieu, it is important to report the morphological adaptations that occurred in order to justify and validate the intervention. Description of subject characteristics, including changes to lower limb lean mass and muscular strength, are provided in Table 1 (available online only), and can be found in the primary manuscript^16^. Briefly, we report, via dual-energy x-ray absorptiometry (DEXA), that significant increases in muscle mass of the lower limb were attained following 7 weeks of training, that returned towards baseline control levels upon detraining. Subsequently, 7-weeks of secondary exposure to resistance exercise (retraining) induced a further increase in muscle mass.

### Quality Control of Isolated DNA

Samples were assessed for quantity and quality of isolated DNA from original muscle samples via UV spectroscopy (Nanodrop 2000, ThermoFisher Scientific, United States), where the mean yield of isolated DNA was 7.8 μg (±4.4 SD) and quality indicated via absorbency at 260/280 UV wavelength was 1.87 (±0.10 SD).

### Quality Control of DNA Methylation Probes

Principal Component Analysis (PCA; Fig. 2f) and frequency plots by lines (Fig. 2g) were performed to detect for probe outliers. Following analysis, two experimental samples (relating to one ‘baseline’ and one ‘unloading’ sample; SkM_Epi_Mem_1 and SkM_Epi_Mem_39, respectively see GSE database and Table 2 for further detail) were identified as outliers and thus were removed from further down-stream analysis. All other samples were deemed sufficient and passed quality control validations. In relation to the heterogeneity of tissue samples, it may be plausible that a small proportion of other non-muscle cells exist in these derived samples. However, PCA analysis suggests sample homogeneity was consistent in the experimental groups and therefore downstream analysis was representative of skeletal muscle tissue and its niche.

## Additional information

**How to cite this article**: Seaborne, R. A. *et al.* Methylome of human skeletal muscle after acute & chronic resistance exercise training, detraining & retraining. *Sci. Data*. 5:180213 doi: 10.1038/sdata.2018.213 (2018).

**Publisher’s note**: Springer Nature remains neutral with regard to jurisdictional claims in published maps and institutional affiliations.

## Supplementary Material



Supplementary Table 1

## Figures and Tables

**Table 1:** Sample information for Methylation EPIC Bead Chip Array in human skeletal muscle following acute resistance (acute RE) exercise, 7-weeks of resistance training (loading), 7-weeks exercise cessation/detraining (unloading) and 7-weeks of re-training (reloading)

Subject	Condition	IDAT File Reference	Source	Sample Name	Method 1	Method 2	Method 3	Data
Participant 1	Baseline (replicate 1)	201496860025_R01C01_Grn.idat201496860025_R01C01_Red.idat	Vastsus Lateralis	SkM_Epi_Mem_1	DNA Isolation	Bisulfite Conversion	MethylationEPICBeadChip Array	GSM3149860
Participant 2	Baseline	201496860025_R06C01_Grn.idat201496860025_R06C01_Red.idat	Vastsus Lateralis	SkM_Epi_Mem_6	DNA Isolation	Bisulfite Conversion	MethylationEPICBeadChip Array	GSM3149865
Participant 3	Baseline	201465940053_R03C01_Grn.idat201465940053_R03C01_Red.idat	Vastsus Lateralis	SkM_Epi_Mem_11	DNA Isolation	Bisulfite Conversion	MethylationEPICBeadChip Array	GSM3149870
Participant 4	Baseline	201465940053_R08C01_Grn.idat201465940053_R08C01_Red.idat	Vastsus Lateralis	SkM_Epi_Mem_16	DNA Isolation	Bisulfite Conversion	MethylationEPICBeadChip Array	GSM3149875
Participant 5	Baseline	201496850072_R05C01_Grn.idat201496850072_R05C01_Red.idat	Vastsus Lateralis	SkM_Epi_Mem_21	DNA Isolation	Bisulfite Conversion	MethylationEPICBeadChip Array	GSM3149880
Participant 6	Baseline	201496860106_R02C01_Grn.idat201496860106_R02C01_Red.idat	Vastsus Lateralis	SkM_Epi_Mem_26	DNA Isolation	Bisulfite Conversion	MethylationEPICBeadChip Array	GSM3149885
Participant 7	Baseline	201496860106_R07C01_Grn.idat201496860106_R07C01_Red.idat	Vastsus Lateralis	SkM_Epi_Mem_31	DNA Isolation	Bisulfite Conversion	MethylationEPICBeadChip Array	GSM3149890
Participant 8	Baseline	201496860128_R04C01_Grn.idat201496860128_R04C01_Red.idat	Vastsus Lateralis	SkM_Epi_Mem_36	DNA Isolation	Bisulfite Conversion	MethylationEPICBeadChip Array	GSM3149895
Participant 1	Acute RE	201496860025_R02C01_Grn.idat201496860025_R02C01_Red.idat	Vastsus Lateralis	SkM_Epi_Mem_2	DNA Isolation	Bisulfite Conversion	MethylationEPICBeadChip Array	GSM3149861
Participant 2	Acute RE	201496860025_R07C01_Grn.idat201496860025_R07C01_Red.idat	Vastsus Lateralis	SkM_Epi_Mem_7	DNA Isolation	Bisulfite Conversion	MethylationEPICBeadChip Array	GSM3149866
Participant 3	Acute RE	201465940053_R04C01_Grn.idat201465940053_R04C01_Red.idat	Vastsus Lateralis	SkM_Epi_Mem_12	DNA Isolation	Bisulfite Conversion	MethylationEPICBeadChip Array	GSM3149871
Participant 4	Acute RE	201496850072_R01C01_Grn.idat201496850072_R01C01_Red.idat	Vastsus Lateralis	SkM_Epi_Mem_17	DNA Isolation	Bisulfite Conversion	MethylationEPICBeadChip Array	GSM3149876
Participant 5	Acute RE	201496850072_R06C01_Grn.idat201496850072_R06C01_Red.idat	Vastsus Lateralis	SkM_Epi_Mem_22	DNA Isolation	Bisulfite Conversion	MethylationEPICBeadChip Array	GSM3149881
Participant 6	Acute RE	201496860106_R03C01_Grn.idat201496860106_R03C01_Red.idat	Vastsus Lateralis	SkM_Epi_Mem_27	DNA Isolation	Bisulfite Conversion	MethylationEPICBeadChip Array	GSM3149886
Participant 7	Acute RE	201496860106_R08C01_Grn.idat201496860106_R08C01_Red.idat	Vastsus Lateralis	SkM_Epi_Mem_32	DNA Isolation	Bisulfite Conversion	MethylationEPICBeadChip Array	GSM3149891
Participant 8	Acute RE	201496860128_R05C01_Grn.idat201496860128_R05C01_Red.idat	Vastsus Lateralis	SkM_Epi_Mem_37	DNA Isolation	Bisulfite Conversion	MethylationEPICBeadChip Array	GSM3149896
Participant 1	Training (loading)	201496860025_R03C01_Grn.idat201496860025_R03C01_Red.idat	Vastsus Lateralis	SkM_Epi_Mem_3	DNA Isolation	Bisulfite Conversion	MethylationEPICBeadChip Array	GSM3149862
Participant 2	Training (loading)	201496860025_R08C01_Grn.idat201496860025_R08C01_Red.idat	Vastsus Lateralis	SkM_Epi_Mem_8	DNA Isolation	Bisulfite Conversion	MethylationEPICBeadChip Array	GSM3149867
Participant 3	Training (loading)	201465940053_R05C01_Grn.idat201465940053_R05C01_Red.idat	Vastsus Lateralis	SkM_Epi_Mem_13	DNA Isolation	Bisulfite Conversion	MethylationEPICBeadChip Array	GSM3149872
Participant 4	Training (loading)	201496850072_R02C01_Grn.idat201496850072_R02C01_Red.idat	Vastsus Lateralis	SkM_Epi_Mem_18	DNA Isolation	Bisulfite Conversion	MethylationEPICBeadChip Array	GSM3149877
Participant 5	Training (loading)	201496850072_R07C01_Grn.idat201496850072_R07C01_Red.idat	Vastsus Lateralis	SkM_Epi_Mem_23	DNA Isolation	Bisulfite Conversion	MethylationEPICBeadChip Array	GSM3149882
Participant 6	Training (loading)	201496860106_R04C01_Grn.idat201496860106_R04C01_Red.idat	Vastsus Lateralis	SkM_Epi_Mem_28	DNA Isolation	Bisulfite Conversion	MethylationEPICBeadChip Array	GSM3149887
Participant 7	Training (loading)	201496860128_R01C01_Grn.idat201496860128_R01C01_Red.idat	Vastsus Lateralis	SkM_Epi_Mem_33	DNA Isolation	Bisulfite Conversion	MethylationEPICBeadChip Array	GSM3149892
Participant 8	Training (loading)	201496860128_R06C01_Grn.idat201496860128_R06C01_Red.idat	Vastsus Lateralis	SkM_Epi_Mem_38	DNA Isolation	Bisulfite Conversion	MethylationEPICBeadChip Array	GSM3149897
Participant 1	Cessation/detraining (unloading)	201496860025_R04C01_Grn.idat201496860025_R04C01_Red.idat	Vastsus Lateralis	SkM_Epi_Mem_4	DNA Isolation	Bisulfite Conversion	MethylationEPICBeadChip Array	GSM3149863
Participant 2	Cessation/detraining (unloading)	201465940053_R01C01_Grn.idat201465940053_R01C01_Red.idat	Vastsus Lateralis	SkM_Epi_Mem_9	DNA Isolation	Bisulfite Conversion	MethylationEPICBeadChip Array	GSM3149868
Participant 3	Cessation/detraining (unloading)	201465940053_R06C01_Grn.idat201465940053_R06C01_Red.idat	Vastsus Lateralis	SkM_Epi_Mem_14	DNA Isolation	Bisulfite Conversion	MethylationEPICBeadChip Array	GSM3149873
Participant 4	Cessation/detraining (unloading)	201496850072_R03C01_Grn.idat201496850072_R03C01_Red.idat	Vastsus Lateralis	SkM_Epi_Mem_19	DNA Isolation	Bisulfite Conversion	MethylationEPICBeadChip Array	GSM3149878
Participant 5	Cessation/detraining (unloading)	201496850072_R08C01_Grn.idat201496850072_R08C01_Red.idat	Vastsus Lateralis	SkM_Epi_Mem_24	DNA Isolation	Bisulfite Conversion	MethylationEPICBeadChip Array	GSM3149883
Participant 6	Cessation/detraining (unloading)	201496860106_R05C01_Grn.idat201496860106_R05C01_Red.idat	Vastsus Lateralis	SkM_Epi_Mem_29	DNA Isolation	Bisulfite Conversion	MethylationEPICBeadChip Array	GSM3149888
Participant 7	Cessation/detraining (unloading)	201496860128_R02C01_Grn.idat201496860128_R02C01_Red.idat	Vastsus Lateralis	SkM_Epi_Mem_34	DNA Isolation	Bisulfite Conversion	MethylationEPICBeadChip Array	GSM3149893
Participant 8	Cessation/detraining (unloading)	201496860128_R07C01_Grn.idat201496860128_R07C01_Red.idat	Vastsus Lateralis	SkM_Epi_Mem_39	DNA Isolation	Bisulfite Conversion	MethylationEPICBeadChip Array	GSM3149898
Participant 1	Retraining (reloading)	201496860025_R05C01_Grn.idat201496860025_R05C01_Red.idat	Vastsus Lateralis	SkM_Epi_Mem_5	DNA Isolation	Bisulfite Conversion	MethylationEPICBeadChip Array	GSM3149864
Participant 2	Retraining (reloading)	201465940053_R02C01_Grn.idat201465940053_R02C01_Red.idat	Vastsus Lateralis	SkM_Epi_Mem_10	DNA Isolation	Bisulfite Conversion	MethylationEPICBeadChip Array	GSM3149869
Participant 3	Retraining (reloading)	201465940053_R07C01_Grn.idat201465940053_R07C01_Red.idat	Vastsus Lateralis	SkM_Epi_Mem_15	DNA Isolation	Bisulfite Conversion	MethylationEPICBeadChip Array	GSM3149874
Participant 4	Retraining (reloading)	201496850072_R04C01_Grn.idat201496850072_R04C01_Red.idat	Vastsus Lateralis	SkM_Epi_Mem_20	DNA Isolation	Bisulfite Conversion	MethylationEPICBeadChip Array	GSM3149879
Participant 5	Retraining (reloading)	201496860106_R01C01_Grn.idat201496860106_R01C01_Red.idat	Vastsus Lateralis	SkM_Epi_Mem_25	DNA Isolation	Bisulfite Conversion	MethylationEPICBeadChip Array	GSM3149884
Participant 6	Retraining (reloading)	201496860106_R06C01_Grn.idat201496860106_R06C01_Red.idat	Vastsus Lateralis	SkM_Epi_Mem_30	DNA Isolation	Bisulfite Conversion	MethylationEPICBeadChip Array	GSM3149889
Participant 7	Retraining (reloading)	201496860128_R03C01_Grn.idat201496860128_R03C01_Red.idat	Vastsus Lateralis	SkM_Epi_Mem_35	DNA Isolation	Bisulfite Conversion	MethylationEPICBeadChip Array	GSM3149894
Participant 1	Baseline (replicate 2)	201496860128_R08C01_Grn.idat201496860128_R08C01_Red.idat	Vastsus Lateralis	SkM_Epi_Mem_40	DNA Isolation	Bisulfite Conversion	MethylationEPICBeadChip Array	GSM3149899

**Table 2:** Sample information for Methylation EPIC Bead Chip Array in human skeletal muscle following acute resistance (acute RE) exercise, 7-weeks of resistance training (loading), 7-weeks exercise cessation/detraining (unloading) and 7-weeks of re-training (reloading).

	**Age**	**Height (cm)**	**Weight (kg)**	**Lean Mass (kg)**				**Isometric Voluntary Contraction (Nm)**			
				**Baseline**	**Training (Loading)**	**Cessation/Detraining (Unloading)**	**Retraining (Reloading)**	**Baseline**	**Training (Loading)**	**Cessation/Detraining (Unloading)**	**Retraining (Reloading)**
Participant 1	39	175	72.8	17.21	18.77	17.62	19.55	223.4	245.2	217.6	257.6
Participant 2	19	182.2	72.2	20.28	22.50	21.20	23.91	336.9	352.6	327.3	399.1
Participant 3	29	177.2	78.6	20.00	21.00	19.88	21.81	274.2	344.1	319.2	370.3
Participant 4	27	196	117.8	24.14	25.60	24.48	26.00	390.0	439.3	364.3	452.6
Participant 5	32	176.4	79.0	19.54	20.77	20.31	21.95	304.6	345.9	349.2	380.5
Participant 6	30	174.4	74.0	19.52	20.48	19.93	22.31	310.6	320.2	315.7	332.5
Participant 7	23	172.0	68.0	15.58	16.12	15.47	17.43	233.3	224.2	219.8	254.4
Participant 8	32	171.5	97.8	16.94	18.64	17.52	–	244.7	224.5	198.8	–
