# Supplementary material for: Methylome of human skeletal muscle after acute & chronic resistance exercise training, detraining & retraining
Source: Sci Data. 2018 Oct 30;5:180213. doi: 10.1038/sdata.2018.213 (PMC6207066; doi:10.1038/sdata.2018.213)
Supplement: Supplementary Table 1 [file sdata2018213-s2.docx]

Supplementary Table 1. Quantity and quality of eluted DNA for all samples used for methylome analysis. All analysis was performed via spectroscopy (Nanodrop 2000, ThermoFisher Scientific, United States).

| **Subject** | **Condition** | **Sample Name** | **ng/μl** | **Quantity (μl)** | **260/280** |
| --- | --- | --- | --- | --- | --- |
| Participant 1 | Baseline  (replicate 1) | SkM_Epi_Mem_1 | 11.9 | 100 | 1.62 |
| Participant 2 | Baseline | SkM_Epi_Mem_6 | 106.5 | 100 | 1.96 |
| Participant 3 | Baseline | SkM_Epi_Mem_11 | 115 | 100 | 1.93 |
| Participant 4 | Baseline | SkM_Epi_Mem_16 | 53.5 | 100 | 1.92 |
| Participant 5 | Baseline | SkM_Epi_Mem_21 | 93.2 | 100 | 1.84 |
| Participant 6 | Baseline | SkM_Epi_Mem_26 | 109.7 | 100 | 1.93 |
| Participant 7 | Baseline | SkM_Epi_Mem_31 | 135.4 | 100 | 1.94 |
| Participant 8 | Baseline | SkM_Epi_Mem_36 | 89.2 | 100 | 1.89 |
| Participant 1 | Acute RE | SkM_Epi_Mem_2 | 76.7 | 100 | 1.89 |
| Participant 2 | Acute RE | SkM_Epi_Mem_7 | 118.8 | 100 | 1.96 |
| Participant 3 | Acute RE | SkM_Epi_Mem_12 | 61.8 | 100 | 1.89 |
| Participant 4 | Acute RE | SkM_Epi_Mem_17 | 129.8 | 100 | 1.96 |
| Participant 5 | Acute RE | SkM_Epi_Mem_22 | 37 | 100 | 1.73 |
| Participant 6 | Acute RE | SkM_Epi_Mem_27 | 35.2 | 100 | 1.73 |
| Participant 7 | Acute RE | SkM_Epi_Mem_32 | 24.5 | 100 | 1.8 |
| Participant 8 | Acute RE | SkM_Epi_Mem_37 | 167.6 | 100 | 1.95 |
| Participant 1 | Training (loading) | SkM_Epi_Mem_3 | 32.6 | 100 | 1.86 |
| Participant 2 | Training (loading) | SkM_Epi_Mem_8 | 65.5 | 100 | 1.93 |
| Participant 3 | Training (loading) | SkM_Epi_Mem_13 | 110 | 100 | 1.93 |
| Participant 4 | Training (loading) | SkM_Epi_Mem_18 | 72.6 | 100 | 1.93 |
| Participant 5 | Training (loading) | SkM_Epi_Mem_23 | 80.2 | 100 | 1.81 |
| Participant 6 | Training (loading) | SkM_Epi_Mem_28 | 54 | 100 | 1.86 |
| Participant 7 | Training (loading) | SkM_Epi_Mem_33 | 59.8 | 100 | 1.89 |
| Participant 8 | Training (loading) | SkM_Epi_Mem_38 | 77.5 | 100 | 1.9 |
| Participant 1 | Cessation/detraining (unloading) | SkM_Epi_Mem_4 | 55.9 | 100 | 1.87 |
| Participant 2 | Cessation/detraining (unloading) | SkM_Epi_Mem_9 | 60.2 | 100 | 1.94 |
| Participant 3 | Cessation/detraining (unloading) | SkM_Epi_Mem_14 | 61 | 100 | 1.92 |
| Participant 4 | Cessation/detraining (unloading) | SkM_Epi_Mem_19 | 50 | 100 | 1.91 |
| Participant 5 | Cessation/detraining (unloading) | SkM_Epi_Mem_24 | 58.9 | 100 | 1.8 |
| Participant 6 | Cessation/detraining (unloading) | SkM_Epi_Mem_29 | 213.6 | 100 | 1.95 |
| Participant 7 | Cessation/detraining (unloading) | SkM_Epi_Mem_34 | 140.7 | 100 | 1.84 |
| Participant 8 | Cessation/detraining (unloading) | SkM_Epi_Mem_39 | 18.3 | 100 | 1.67 |
| Participant 1 | Retraining (reloading) | SkM_Epi_Mem_5 | 64 | 100 | 1.92 |
| Participant 2 | Retraining (reloading) | SkM_Epi_Mem_10 | 112.9 | 100 | 1.93 |
| Participant 3 | Retraining (reloading) | SkM_Epi_Mem_15 | 67.3 | 100 | 1.91 |
| Participant 4 | Retraining (reloading) | SkM_Epi_Mem_20 | 25.7 | 100 | 1.83 |
| Participant 5 | Retraining (reloading) | SkM_Epi_Mem_25 | 61.1 | 100 | 1.87 |
| Participant 6 | Retraining (reloading) | SkM_Epi_Mem_30 | 104.3 | 100 | 1.9 |
| Participant 7 | Retraining (reloading) | SkM_Epi_Mem_35 | 139.7 | 100 | 1.93 |
| Participant 1 | Baseline  (replicate 2) | SkM_Epi_Mem_40 | 13.8 | 100 | 1.53 |
